# The Capabilities of Chaos and Complexity

**DOI:** 10.3390/ijms10010247

**Published:** 2009-01-09

**Authors:** David L. Abel

**Affiliations:** The Gene Emergence Project, The Origin of Life Science Foundation, Inc. 113–120 Hedgewood Dr. Greenbelt, MD 20770-1610 USA. E-Mail: life@us.net; Tel. 301-441-2923; Fax: 301-441-8135

**Keywords:** Complex adaptive systems (CAS), Complexity theory, Biocybernetics, Biosemiotics, Emergence, Non linear dynamics, Self-organization, Symbolic dynamics analysis, Systems theory

## Abstract

To what degree could chaos and complexity have organized a Peptide or RNA World of crude yet necessarily integrated protometabolism? How far could such protolife evolve in the absence of a heritable linear digital symbol system that could mutate, instruct, regulate, optimize and maintain metabolic homeostasis? To address these questions, chaos, complexity, self-ordered states, and organization must all be carefully defined and distinguished. In addition their cause-and-effect relationships and mechanisms of action must be delineated. Are there any formal (non physical, abstract, conceptual, algorithmic) components to chaos, complexity, self-ordering and organization, or are they entirely physicodynamic (physical, mass/energy interaction alone)? Chaos and complexity can produce some fascinating self-ordered phenomena. But can spontaneous chaos and complexity steer events and processes toward pragmatic benefit, select function over non function, optimize algorithms, integrate circuits, produce computational halting, organize processes into formal systems, control and regulate existing systems toward greater efficiency? The question is pursued of whether there might be some yet-to-be discovered new law of biology that will elucidate the derivation of prescriptive information and control. “System” will be rigorously defined. Can a low-informational rapid succession of Prigogine’s dissipative structures self-order into bona fide organization?

## 1. Introduction

Stand-alone chaos, complexity and catastrophe should never be confused with our theories and what we intelligent humans do using abstract conceptual nonlinear dynamic models. Life-origin science is not especially interested in:

1. Modern-day human applications of non linear dynamical systems theory.
2. Investigator involvement (artificial selection) in chaos, catastrophe, and complexity experimental designs.
3. Information defined in terms of the reduced uncertainty of subjective “observers” and “knowers”, who did not exist for 99.9% of life’s history.

Life origin science wants to know the capabilities of stand-alone chaos and complexity before any animal consciousness existed. If all known life depends upon genetic instructions, how was the first linear digital prescriptive genetic information generated by natural process? In the absence of human thought and involvement, can objective chaos, complexity and catastrophe generate either or both of two peculiar entities:

1. Prescriptive Information (PI) [1–3]? PI refers not just to intuitive or semantic information, but specifically to linear digital instructions using a symbol system (e.g., 0’s and 1’s, letter selections from an alphabet, A, G, T, or C from a phase space of four nucleotides). PI can also consist of purposefully programmed configurable switch-settings that provide cybernetic controls.
2. *Bona fide* Formal Organization [4]? By “formal” we mean function-oriented, computationally halting, integrated-circuit producing, algorithmically optimized, and choice-contingent at true decision nodes (not just combinatorial bifurcation points).

Both PI and formal organization are abstract, conceptual, non physical entities [1–13]. Scientific endeavors to better understand cybernetic reality in nature are confronted with the uneasy suggestion of its transcendence over the physicality it controls. At the heart of all naturalistic life-origin models lies the presumption of self-organization of inanimate physicality into sophisticated formal utility. The notion of *emergence* can be traced back to Aristotle [14], but George H. Lewes was probably the first to define it in 1875: “The emergent is unlike its components insofar as these are incommensurable, and it cannot be reduced to their sum or their difference.” [15. pg. 412]. The idea of emergence blossomed in the 1920’s with contributions from C. Lloyd Morgan, C. D. Broad, Samuel Alexander, Henre Bergson, Alfred North Whitehead, and Arthur O. Lovejoy [16]. Weak and strong versions of emergence exist [17], but life-origin models of necessity require convincing models of strong emergence. The whole is greater than the sum of its parts [18]. Novel functional qualities are believed to arise spontaneously from inanimate physical components [19–22]. First, second, third and now fourth order (Types I–IV) emergence are said to exist [23]. Heritable linear digital genetic prescription can produce three-dimensional protein molecular machines that bind, transport and catalyze metabolic integration. Strong and Type IV emergent theory together attempt to explain the source of these phenomena. Admits Mark Bedau, “Although strong emergence is logically possible, it is uncomfortably like magic.” [24].

If Pasteur and Virchow’s First Law of Biology (“All life must come from previously existing life”) is to be empirically falsified, direct observation of spontaneous generation is needed. In the absence of such empirical falsification, a plausible model of mechanism at the very least for both Strong and Type IV emergence (formal self-organization) is needed. Manfred Eigen [25–36] and Tibor Ganti [37–41] have been leaders in the search for mechanisms of biologic emergence from abiotic environments. Shuster joined with Eigen to hypothesize hypercycles [42–49]. The Edge of Chaos [21, 22, 50–57] has been proposed as a possible source, though the description of all of the above models often seems more poetic or cartoon-like than real. Kauffman’s and Dawkin’s publications, for example, are often devoid of any consideration of the biochemical catastrophic realities that plague life-origin bench scientists [20–22, 58–63].

Attempts to define complexity are on-going [50, 64–70]. Sequence complexity has been extensively studied, though far from exhaustively [1, 71–77].

Much debate has occurred over the relation of linear complexity to semantic information [78–83] [84–92]. Some have attempted to reduce the information of linear digital prescription in genes to mere thermodynamics, combinatorial probabilism, and physicodynamic complexity [20, 93–106]. Other investigators tend to view genetic information as literal and real [1, 2, 6, 107–112]. The special case of semiotic linear digital complexity has fostered the whole new field of Biosemiotics [2, 113–133].

The cause and evolution of complexity are frequently addressed in the literature [10, 134–141]. How complexity relates to life has attracted innumerable papers [6, 142–148]. Systems Biology emphasizes the growing genomic and epigenetic complexity [149–151]. Attempts to deal with Behe’s “irreducible complexity” [152] are appearing more often in scientific literature [153–157]. von Neumann [158] and Pattee [159–161] attempted to deal with the issue of Complementarity between the formal and physical aspects of complexity. Hoffmeyer and Emmeche have addressed the same basic problem with Code Duality [162, 163]. Stein described the different sciences of complexity [164]. Norris has researched hypercomplexity [165]; Garzon dealt with bounded complexity [166]; and Levins the limits of complexity [167]. Bennett originated Logical Depth and its relation to physical complexity [168].

Attempts to relate complexity to self-organization are too numerous to cite [4, 21, 169–171]. Under careful scrutiny, however, these papers seem to universally incorporate investigator agency into their experimental designs. To stem the growing swell of Intelligent Design intrusions, it is imperative that we provide *stand-alone natural process* evidence of non trivial self-organization at the edge of chaos. We must demonstrate on sound scientific grounds the formal capabilities of naturally-occurring physicodynamic complexity. Evolutionary algorithms, for example, must be stripped of all artificial selection and the purposeful steering of iterations toward desired products. The latter intrusions into natural process clearly violate sound evolution theory [172, 173]. Evolution has no goal [174, 175]. Evolution provides no steering toward *potential* computational and cybernetic function [4, 6–11].

The theme of this paper is the active pursuit of falsification of the following null hypothesis: “Physicodynamics alone cannot organize itself into formally functional systems requiring algorithmic optimization, computational halting, and circuit integration.” At first glance the falsification of this hypothesis might seem like a daunting task. But a single exception of non trivial, unaided, spontaneous optimization of formal function by truly natural process would quickly falsify this null hypothesis.

Science celebrates positive and parsimonious descriptions of presumed objectivity. But we must never forget that our knowledge is only “best thus far.” Even the most fundamental laws of physics technically must be viewed as “tentative.” We rightly eschew diatribes of metaphysical pontifications. Science proceeds through open-mindedness and the falsification of null hypotheses, not through the rhetorical pronouncement of dogmas. Popper and many since have exposed the problems associated with trying to prove any positive hypothesis [176, 177]. Neither induction nor deduction is foolproof. Theses that cannot be proven ought not to be proclaimed as positive statements of fact.

At the same time, we have spent much of the last century arguing to the lay community that we *have* proved the current biological paradigm. Unfortunately, very few in the scientific community seem critical of this indiscretion. One would think that if all this evidence is so abundant, it would be quick and easy to falsify the null hypothesis put forward above. If, on the other hand, no falsification is forthcoming, a more positive thesis might become rather obvious by default. Any positive pronouncement would only be labeled metaphysical by true-believers in spontaneous self-organization. Those same critics would disingenuously fail to acknowledge the purely metaphysical nature of the current Kuhnian paradigm rut [178]. A better tact is to thoroughly review the evidence. Let the reader provide the supposedly easy falsification of the null hypothesis. Inability to do so should cause pangs of conscience in any scientist who equates metaphysical materialism with science. On the other hand, providing the requested falsification of this null hypothesis would once-and-for-all end a lot of unwanted intrusions into science from philosophies competing with metaphysical materialism.

While proof may be evasive, science has an obligation to be honest about what the entire body of evidence clearly *suggests*. We cannot just keep endlessly labeling abundant evidence of formal prescription in nature “apparent.” The fact of purposeful programming at multiple layers gets more “apparent” with each new issue of virtually every molecular biology journal [179–181]. Says de Silva and Uchiyama:

Molecular substrates can be viewed as computational devices that process physical or chemical ‘inputs’ to generate ‘outputs’ based on a set of logical operators. By recognizing this conceptual crossover between chemistry and computation, it can be argued that the success of life itself is founded on a much longer-term revolution in information handling when compared with the modern semiconductor computing industry. Many of the simpler logic operations can be identified within chemical reactions and phenomena, as well as being produced in specifically designed systems. Some degree of integration can also be arranged, leading, in some instances, to arithmetic processing. These molecular logic systems can also lend themselves to convenient reconfiguring. Their clearest application area is in the life sciences, where their small size is a distinct advantage over conventional semiconductor counterparts. Molecular logic designs aid chemical (especially intracellular) sensing, small object recognition and intelligent diagnostics [181].

What scientific evidence exists of physicodynamics ever having programmed a single purposeful configurable switch-setting? If we cannot present any such evidence, we should be self-honest enough to start asking ourselves, “How long are we going to try to maintain this ruse that the cybernetic programming we repeatedly observe is only ‘apparent’ rather than real?”

## 2. What exactly is complexity?

“Complexity” can tend to be a garbage-can catch-all term we use to explain everything we don’t understand and cannot reduce. To define complexity, we need to start with one dimension and work up. When we progress from linear complexity into two and three dimensional complexity, quantifying the degree of complexity can quickly become intractable [182]. Thus, let us begin by precisely defining linear sequence complexity.

An unequivocal, pristine, mathematical definition of linear “complexity” already exists in scientific literature [1, 71, 183]: maximum complexity in a linear string, oddly enough, is randomness. Maximum complexity cannot be compressed because it lacks patterns and order [183, 184]. A random string (Random Sequence Complexity, RSC) [1] is the most complex because its sequence cannot be enumerated using any algorithmically compressive string shorter than itself. Notice that this precise definition of linear complexity has nothing to do with meaning or function. Complexity in linear digital strings is fully measurable by the degree to which each string can be algorithmically compressed. This is true whether the string does anything useful or not. A string comprised of maximally uncertain elements will be the most complex string because it lacks order and pattern. The compressibility of that string is therefore extremely low. Uncertainty is measured in bits. The higher the number of bits of uncertainty, the greater the degree of complexity, and the closer we move toward a random string:

(1) $$H=\sum_{i=1}^{M}p_{i}(-\log_{2}p_{i})$$

This of course is Shannon’s basic measurement of uncertainty in linear sequence complexity.

We have invested so much confidence and anticipation in “complexity” as a potential source of spontaneous prescriptive information and organization that our senses are jolted by the pristine mathematical definition of sequence complexity reviewed above. We need to re-educate ourselves with the realization that maximum complexity is nothing more than randomness. The most complex of all strings is a random string. Random strings have never been observed to generate non trivial formal function of any kind. Complexity, therefore, has nothing to do with formal function. Complexity possesses no creative or computational talents. No justification exists for attributing exquisite formal organization to mere complexity.

## 3. Order, structure and pattern

Well, what about order and pattern? If complexity itself is not what produces utility in a linear digital string, surely order, structure and pattern can. But do they? The answer is no! To understand why, we must also define order and pattern.

**Figure 1.**
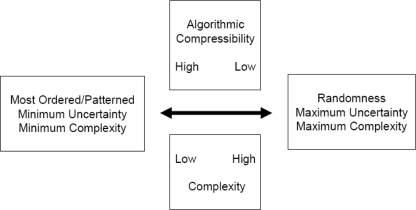
An antithetical relationship exists between linear sequence order and complexity. Randomness affords the greatest measure of complexity. The more ordered and patterned a sequence, the less uncertain are its components, and the less complex the sequence. Neither order nor complexity generates formal meaning or utility, both of which lie in a completely different dimension from order/complexity measures.

What exactly is “order”? Starting with a single dimension, order in a sequence is defined by an increasing probability of occurrence of each structure, event, or alphabetical character in that string [183]. High probability is high order [185]. As the probability of an event approaches 1.0, its Shannon uncertainty approaches 0 bits [186]. 0 bits of uncertainty is maximum order. Maximum order is minimal complexity. Order and complexity are antithetical. They lie at opposite extremes of a bidirectional vector (Figure 1) [71]. The relationship between order and complexity has been well-defined in the literature [1, 3, 71, 187]. Figure 1 shows the antithetical relationship between order and complexity. Order lies on the opposite end of this bidirectional vector graph from complexity. The more complex a sequence is, the less ordered. The more ordered a sequence is, the less complex. The literature is filled with misunderstanding of the relationship between pattern and complexity.

Ordered strings contain repeating patterns such as those found in sugar molecules. As we add dimensions, high order can be found in sine waves and inorganic crystals. Repeating patterns generate high order and low complexity. The probability of encountering the next element of a repeating pattern is high; the probability of coming across any uniqueness (e.g., a crystal impurity) is low.

Highly ordered/patterned strings can be greatly compressed algorithmically. The most ordered string is exampled by a string of identical letters, or a DNA homopolymer consisting of all adenosines. A polyadenosine has maximum order, no uncertainty, and therefore no complexity. A polymer of 200 adenosines can be fully enumerated by the very short compression algorithm, “Give me an adenosine; repeat 200 times.” This compression algorithm for a polyadenosine contains virtually no uncertainty, and therefore no information potential. It is an example of Ordered Sequence Complexity (OSC) [1].

Note that statistical order and pattern have no more to do with function and formal utility than does maximum complexity (randomness). Neither order nor complexity can program, compute, optimize algorithms, or organize.

A law of physics also contains very little information because the data it compresses is so highly ordered. The best way to view a parsimonious physical law is as a compression algorithm for reams of data. This is an aspect of valuing Occam’s razor so highly in science. Phenomena should be explained with as few assumptions as possible. The more parsimonious a statement that reduces all of the data, the better [188, 189]. A sequence can contain much order with frequently recurring patterns, yet manifest no utility. Neither order nor recurring pattern is synonymous with meaning or function.

Those trained in information theory will be quick to point out at this point that “information is always defined in terms of an observer or knower.” They argue that information is not in the law’s parsimonious statement or equation, but in the difference (R) between all of the uncertainty of the raw data, and the lesser amount of uncertainty generated by knowing the law. The problem with this concept of information is that for most of life’s history, linear digital genetic instructions have been prescribing exquisite metabolic organization long before any observers or knowers existed. Observers and knowers themselves would not exist except for the extraordinary amount of cellular programming and organization that produced humans. Prescriptive Information (PI) [3] cannot be reduced to human epistemology. To attempt to define information solely in terms of human observation and knowledge is grossly inadequate. Such anthropocentrism blinds us to the reality of life’s *objective* genetic programming, regulatory mechanisms, and biosemiosis using symbol systems [2, 112, 120, 125, 132, 190–194].

Well what about a combination of order *and* complexity? Doesn’t that explain how prescriptive information comes into being?

Three subsets of linear complexity have been defined in an abiogenesis environment [1]. These subsets are very helpful in understanding potential sources of Functional Sequence Complexity (FSC) as opposed to mere Random Sequence Complexity (RSC) and Ordered Sequence Complexity (OSC) [1]. FSC requires a third dimension not only to detect, but to produce formal utility. Neither chance nor necessity (nor any combination of the two) has ever been observed to produce non trivial FSC [4].

Durston and Chiu at the University of Guelph developed a method of measuring what they call *functional uncertainty* (H_f_) [195]. They extended Shannon uncertainty to measure a *joint variable* (*X, F*), where *X* represents the variability of data, and *F* its functionality. This explicitly incorporated the empirical knowledge of embedded function into the measure of sequence complexity:

(2) $$H(X_{\text{f}}(t))=-\Sigma P(X_{\text{f}}(t))\log P(X_{\text{f}}(t))$$

where *X*_f_ denotes the conditional variable of the given sequence data (*X*) on the described biological function *f* which is an outcome of the variable (*F*). The state variable *t,* representing time or a sequence of ordered events, can be fixed, discrete, or continuous. Discrete changes may be represented as discrete time states. Mathematically, the above measure is defined precisely as an outcome of a discrete-valued variable, denoted as *F=*{*f*}. The set of outcomes can be thought of as specified biological states.

Using this method allowed Durston and Chiu to compare quantifications of 2,442 aligned sequences of proteins belonging to the Ubiquitin protein family, among many other protein families evaluated. All of these sequences satisfied the same specified function *f*, which might represent the known 3-D structure of the Ubiquitin protein family, or some other function common to ubiquitin. The definition of functionality used by Durston and Chiu relates to the whole protein family. Thus this data can be inputted from readily available databases. Even subsets (e.g., the active sites) of the aligned sequences all having the same function can be quantified and compared. The tremendous advantage of using *H*(*X*_f_(*t*)) is that *slight changes* in the functionality characteristics of biosequences can be incorporated and analyzed.

Subsequently, Durston and Chiu have developed a theoretically sound method of actually quantifying Functional Sequence Complexity (FSC) [77]. This method holds great promise in being able to measure the increase or decrease of FSC through evolutionary transitions of both nucleic acid and proteins. This FSC measure, denoted as *ζ,* is defined as the change in functional uncertainty from the ground state *H*(*X*_g_(*t*_i_)) to the functional state *H*(*X*_f_(*t*_i_)), or

(3) $$\zeta =\Delta H(X_{\text{g}}(t_{\text{i}}),\;X_{\text{f}}(t_{\text{j}}))$$

The *ground state g* of a system is the state of presumed highest uncertainty permitted by the constraints of the physical system, when no specified biological function is required or present. Durston and Chiu wisely differentiate the ground state *g* from the *null state H_ø_*. The null state represents the absence of *any* physicodynamic constraints on sequencing. The null state produces bona fide stochastic ensembles, the sequencing of which was *dynamically inert* (physicodynamically decoupled or incoherent [196, 197]).

The FSC variation in various protein families, measured in Fits (Functional bits), is shown in Table 1 graciously provided here by Durston and Chiu. In addition to the results shown in Table 1, they performed a more detailed analysis of ubiquitin, plotting the FSC values out along its sequence. They showed that 6 of the 7 highest value sites correlate with the primary binding domain [77].

**Figure 2.**
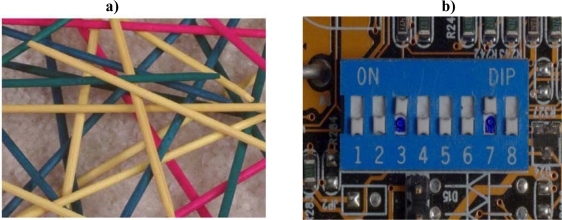
a) The degree of three-dimensional computational complexity within a pile of pick-up sticks is staggering. But what exactly does this enormous degree of complexity DO? What sophisticated formal function does this pile of objects generate? Mere combinatorial complexity must never be confused with formal utility. b) A row of dip switch settings depicts a different category of complexity—algorithmic, cybernetic programming complexity. Choice contingency is incorporated into purposeful configurable switch-settings that collectively prescribe formal function.

In the pile of “Pick-up Sticks” seen in Figure 2, very little order and patterning are present. Uncertainty as to how the sticks will fall is high. The pile of sticks is highly complex. In this three-dimensional model, it would probably be intractable to compute the complexity of relationships of each of these sticks to all of the other sticks. The degree of complexity would be staggering. But what exactly does this enormous degree of complexity DO? The pile of pick-up sticks achieves no utility of any kind. The imagined capabilities of stand-alone complexity are in reality miniscule at best.

Attributing organization to chaos and complexity employs a combination of fallacious inferences involving category errors and non sequiturs. “The edge of chaos” [21, 22, 50–54] affords mesmerizing visions of potential accomplishment. While poetic and wonderfully inviting, the concept is sorely lacking in scientific content. The functional reality of “the edge of chaos” has been challenged [7, 8, 57, 198].

The association of complexity *or* patterns with most forms of bona fide organization should never be confused with causation [199]. Neither order nor complexity is a cause of organization or any other form of formal algorithmic optimization. We sling the words “chaos,” “complexity,” “order” and “pattern” around with vivid imagination and a great deal of blind faith in their capabilities. None of the latter states has ever been observed to produce the slightest amount of algorithmic organization. Stand-alone chaos and complexity have absolutely nothing to do with generating formal function. Neither do order and pattern. Self-ordering phenomena produce boring, unimaginative redundancy. Self-ordering phenomena, just like chaos and complexity, have never been observed to achieve 1) programming, 2) computational halting, 3) creative engineering, 4) symbol systems, 5) language, or 6) bona fide organization [4]. The latter are all formal processes, not physicodynamic processes.

Suppose stochastic ensembles of oligoribonucleotides were forming out of sequence space in an imagined “primordial soup.” Since only 4 different nucleotides could be added next to a forming single positive strand, M in Equation 1 above would = 4. Suppose next that the prebiotic availability p_i_ for adenine was 0.46, and the p_i_ ‘s for uracil, guanine, and cytosine were 0.40, 0.12, and 0.02 respectively. This is being generous for cytosine, since cytosine would have been extremely difficult to make in any prebiotic environment [200]. Using these hypothetical base-availability probabilities, the Shannon uncertainty would have been equal to:

**Table:** 

Adenine	0.46 (– log_2_ 0.46)	= 0.515
Uracil	0.40 (– log_2_ 0.40)	= 0.529
Guanine	0.12 (– log_2_ 0.12)	= 0.367
Cytosine	0.02 (– log_2_ 0.02)	= 0.113
	1.00	1.524 bits

Notice how unequal availability of the four nucleotides (*a form of ordering*) greatly reduces Shannon uncertainty at each locus, and in the entire sequence, of any biopolymeric stochastic ensemble (Figure 1). Maximum uncertainty would occur if all four base availability probabilities were 0.25. Under these equally available base conditions, Shannon uncertainty would have equaled 2 bits per independent nucleotide addition to the strand. A stochastic ensemble formed under aqueous conditions of mostly adenine availability, however, would have had little information-retaining ability because of its high order [1].

As pointed out in the above reference, even less information-retaining ability would be found in an oligoribonucleotide adsorbed onto montmorillonite [201–206]. Clay surfaces would have been required to align ribonucleotides with 3’ 5’ linkages. The problem is that only polyadenosines or polyU’s tend to form. Using clay adsorption to solve one biochemical problem creates an immense informational problem (e.g., high order, low complexity, low uncertainty, and low information retaining ability. See Figure 1). High order means considerable compressibility. The Kolmogorov [207] algorithmic compression program for clay-adsorbed biopolymers (Figure 2) would read: “Choose adenosine; repeat the same choice fifty times.” Such a redundant, highly-ordered sequence could not begin to prescribe even the simplest protometabolism. Such “self-ordering” phenomena would not be the key to life’s early algorithmic programming.

The RNA Word and pre-RNA World models [208, 209] still prevail despite daunting biochemical problems. Life origin models also include clay life [210–213]; early three-dimensional “genomes” [214, 215]; “Metabolism/Peptide First” [216–219]; “Co-evolution” [220–223]; “Simultaneous nucleic acid and protein” [224–226]; and “Two-Step” models of life-origin [227–229]. In virtually all of these life origin models, “self-ordering” is confused with “self-organizing.” No mechanism is provided for the development of a linear digital prescription and oversight system to integrate metabolism. No known life form exists that does not depend upon such genetic instruction.

## 4. Autopoesis

Umberto Maturana and Francisco Varela [230–232] argue for a concept of autopoeisis that presupposes (or begins with) one-celled organisms and progresses evolutionarily all the way up to humans, their language, and their social structure. They used the term autopoiesis to characterize the nature of living systems more than to theorize how cellular life came into existence. An autopoietic system is self-sustaining, homeostatic and autonomous despite having a continuous flow of mass and energy through the cell. Maturana and Varela’s basic contention is that organisms are inherently compelled to maintain their own inner nature and identity. Such a concept of “self-making” might better be described as “self-maintaining.” It does not address the problem of abiogenesis—the spontaneous generation of life from non life at the molecular evolutionary level. Says Varela (who often writes jointly with Maturana),

If living systems are machines, that they are physical autopoietic machines is trivially obvious: they transform matter into themselves in a manner such that the product of their operation is their own organization. However, we deem the converse as also true: A physical system if autopoietic is living. In other words, we claim that the notion of *autopoiesis is necessary and sufficient to characterize the organization of living systems.*

So far as abiogenesis research is concerned, such a statement seems circular or tautological in nature. Their publications offer no mechanisms or help in understanding the processes by which inanimate physics and chemistry wrote life’s cybernetic programming. They do not address how an abiotic physicochemical environment organized chemical reactions into an 11-step biochemical pathway such as the Krebs Cycle. The latter yields no pragmatic benefit until the final biochemical step.

Pier Luigi Luisi, one of the world’s leading experts in primordial membrane theory, points out that Maturana and Valari’s concept of autopoiesis “is not a theory about the origin of life—but rather a pragmatic blueprint of life based on cellular life.” [233] Luigi goes on to state that Maturana and Varela’s theory of autopoiesis “had, and still has, a difficult time being accepted into the mainstream of life-science research” [233].

Margaret Boden [234–236] also tends to presuppose organization rather than to eludicate mechanisms of its abiotic derivation. She challenges Maturana’s and Varela’s use of cognitive language as being too liberal: “Life does not imply cognition.” But many of Boden’s publications themselves presuppose and incorporate human cognition and epistemology into her models. None of these authors purport to offer explanations for life origin. They are simply not on the forefront of abiogenesis research at the biochemical level. No physicochemical mechanism is provided for self-organization.

The term autopoesis has been used on occasion in a much broader sense than Maturana and Varela coined it. Some life-origin investigators use “autopoesis” to refer to the prebiotic “self-making from scratch” of life—of biochemical abiogenesis. But the use of this broader term in some publications has not afforded any new purely physical models of “self-organization” or “emergence”.

## 5. Complex adaptive systems (CAS)

Complex adaptive systems (CAS) [137, 237, 238] are comprised of multiple interconnected (yet diverse) components. CAS readily undergo change. Healthy (CAS) are high-dimensional with respect to turbulence and potential for change. Rigid, low dimensional systems are said to be unhealthy. CAS are called “adaptive” because they are said to learn from experience. Cells, embryos, immune systems, central nervous systems, ecosystems, social insect colonies, human social systems, and economics, are all included in CAS studies. Either the CAS is itself alive, or it is a robot programmed (cybernetically determined) by life to “learn.” It is not surprising that so many systems theory and CAS models presuppose and use life rather than explain the derivation of life. Already-existing cellular prescriptive information is incorporated into the model in virtually every CAS discipline. Self-organization is claimed, but never empirically demonstrated independent of experimenter steering.

Artificial life efforts generally pursue CAS models [6, 146, 239–246] in what amounts to an engineering context. So-called “evolutionary algorithms” and “directed evolution” strategies are often used to support an evolutionary paradigm. But both strategies amount to artificial selection, not natural selection. They have little or nothing to do with neoDarwinism.

Positive and negative feedback plays a major role in systems theory, especially in the social sciences [247–249]. Feedback mechanisms are characterized by a circularity of causation. Output components are fed back into the input. But feedback mechanisms need formal controls to generate sophisticated utilitarian results. Purely physicodynamic hypercycles [44, 47, 49], for example, consume all available resources in their redundant and unimaginative mutual replications. The result is catastrophic with regard to any formal self-organization. Empirical evidence is sorely lacking to support the hoped-for relentless growth in integrated protometabolic function [4]. The excitement that hypercycle theory generated in the 1980’s has proven over time to be little more than Freudian wish fulfillment.

Trying to explain the spontaneous occurrence of CAS encounters early roadblocks. As with Shannon “information” theory, the *potential* information provided by uncertainty measures is not the same as intuitive information [250, 251], semantic information [252–258], biological information [98, 99, 101, 111, 190, 259–268], functional information [269, 270], or the programmed prescriptive information (PI) that generates formal utility [1, 3, 12]. Combinatorial uncertainty is essential in any physical matrix for that matrix to be able to retain instantiated prescriptive information. But mere combinatorial uncertainty and the potential for change possess no programming talents [3, 9, 10].

For complex adaptive systems to progress in the direction of achieving formal utility, selection for potential function must take place at individual decision nodes, logic gates, and configurable switch settings prior to the realization of that function. An inanimate environment cannot do this. In addition, the syntax of such choices for utility must be integrated into programmable circuits to achieve computational halting. Combinatorial uncertainty does not provide any mechanism for steering physicality toward abstract, conceptual, formal computational success. Inanimate nature possesses no motivation, let alone formal skills, to pursue integration of pathways and cycles into a holistic metabolic scheme.

The illusion of abundant empirical support for the spontaneous generation of CAS arises from the conflation of physical combinatorial uncertainty with the agency of the experimenter. The experimenter invariably steers events toward desired function behind the scenes. Even before critiquing Materials and Methods of many CAS and systems theory papers, investigator involvement in experimental design is usually apparent right from the author’s own words. For example, in a paper on adaptive feedback control for linearizable chaotic systems [271] we read: “A remarkable feature of *the proposed approach* is that *it can be used* for chaos control as well as chaos synchronization.” [italics mine] Note the passive voice. Used by what or by whom? “*Numerical simulations* of two well-known chaotic systems are illustrated to show the effectiveness and robustness of the proposed adaptive *control strategy.*” [italics mine] Inanimate nature does not do “numerical simulations.” Where exactly within natural physicodynamic interactions did “control *strategy*” come from? Such steering strategy toward utilitarian capability did not arise from physicodynamics. The steering arose purely from the experimenter’s choice contingency—from the investigator’s goals, experimental design, and artificial selection for what was wanted. Many papers actually acknowledge up front the role of “engineering,” sometimes right in their titles [272–279] (e.g., “Simulation-Based Engineering Of Complex Adaptive Systems” [280]).

Attempts are usually made to attribute this acknowledged need for engineering to evolution [273, 277, 281, 282]. But natural selection never works at the decision node programming level [10]. Evolution works only on already-programmed, already-living, already-fittest phenotypic organisms [175]. Selection pressure is nothing more than the differential survival and reproduction of the fittest small populations of living organisms [10]. An adequate selection mechanism for *potential* computational function has always been lacking in evolution theory. This is all the more painfully apparent at the molecular evolution level. No basis for preferring stand-alone function over non function exists in an inanimate prebiotic environment [10]. Worse yet, an inanimate environment has no ability to program for a *potential* function that does not yet exist [9]. Yet selection for potential function is exactly what genetic programming requires [12]. Genetic programming is “written in stone” into linear digital sequences bound by rigid 3’5’ phosphodiester covalent bonds prior to transcription, translation, protein-folding, and three-dimensional metabolism [3].

## 6. The big three: Chance, necessity and selection

Selection must be included along with “chance and necessity” [174] as a fundamental category of reality. Why? First, biological science presupposes natural selection as its single most organizing paradigm. Without *selection*, evolution is impossible. Linear digital genetic instructions represent *selection-based* cybernetic programming. Life uses a symbol system as evidenced by the codon table. Symbols must be selected from an alphabet of symbols. Nucleotides must be selected from a phase space of four options at each locus in the DNA string. Second, the scientific method pre-assumes the reality and reliability of formal rationality, mathematics, cybernetic programming, and predictive computations. All of these operational tools depend upon decision theory [283–285]. The practice of science would be impossible without selection at bona fide decision nodes, logic gates and configurable switch settings. Chance and necessity, as Monod pointed out [174], are inadequate to describe everything we repeatedly observe, life especially. Science must acknowledge the reality and validity of selection as a fundamental, “properly basic” category.

A third dimension is required to see what relation, if any, order and complexity have to meaning and function. It is possible to write a highly functional computationally halting program that is non compressible (manifests no patterns). Each symbol selection represents an independent choice. A third dimension is required to distinguish what appears to be a random string from a functional program. That third dimension is the reality of selection, whether in the form of a) natural selection, or b) artificial selection:

- Natural selection is a very special case indeed. Differential survival and reproduction of the fittest already-computed, already-living small populations of organisms is very indirect. Selection is not intended; it just happens secondarily. No purpose guides selection events. No true decision nodes are involved because evolution has no goal. In this sense, selection “pressure” is a misnomer. Differential survival is more happenstantial than pushed, more after-the-fact than pursued.
- Artificial selection is the essence of formalism. Despite decades of concentrated research on consciousness and artificial intelligence, choice contingency remains elusive when approached from the direction of physicality. The mind/body problem is alive and well in the philosophy of science.

No known natural process exists that spontaneously writes meaningful or functional syntax. Only agents have been known to write meaningful and pragmatic syntax. Physicality cannot compute or make arbitrary symbol selections according to arbitrarily written rules. Physicality cannot compress. Physicality cannot value or pursue formal utility. Physicality is blind to pragmatic considerations, all of which are formally valued and pursued. No known mechanism exists in inanimate nature to steer physical events toward algorithmic optimization. Many epigenetic factors notwithstanding, genetics and genomics largely *program* phenotypes using a symbol system of linear digital prescription.

## 7. Do symbol systems exist outside of human minds?

Metabolism employs primarily proteins. The nucleotide sequences in mRNA prescribe the amino acid sequences that determine protein identity. DNA is largely inert. It plays no direct physicochemical role in protein binding, transport and catalysis. Molecular biology’s two-dimensional complexity (secondary biopolymeric structure) and three-dimensional complexity (tertiary biopolymeric structure) are both ultimately determined by linear sequence complexity (primary structure; functional sequence complexity, FSC). The chaperone proteins that aid polyamino acid folding are also prescribed by the linear digital genetic programming instantiated into DNA sequencing.

Genetics not only utilizes a linear digital symbol system, but abstract Hamming block coding to reduce noise pollution in the Shannon channel (triplet codons to prescribe each amino acid). Anti-codons are at opposite ends of t-RNA molecules from amino acids. The linking of each tRNA with the correct amino acid depends entirely upon on a completely independent family of tRNA aminoacyl synthetase proteins. Each of these synthetases must be specifically prescribed by separate linear digital programming, but using the same MSS. These symbol and coding systems not only predate human existence, they *produced* humans along with their anthropocentric minds. The nucleotide and codon syntax of DNA linear digital prescription has no physicochemical explanation. All nucleotides are bound with the same rigid 3’5’ phosphodiester bonds. The codon table is arbitrary and formal, not physical. The semantic/semiotic/bioengineering function required to make proteins requires dynamically inert configurable switch-settings and resortable physical symbol vehicles. Codon syntax communicates time-independent, non-physicodynamic “meaning” (prescription of biofunction). This meaning is realized only after abstract translation via a conceptual codon table. To insist that codon syntax only represents amino acid sequence in our human minds is not logically tenable.

**Figure 3.**
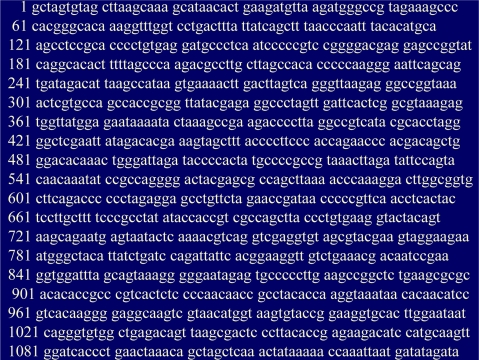
A section of *Alosa pseudoharengus* (a fish) mitochondrion DNA. This reference sequence continues on all the way up to 16,621 “letters.” Each nucleotide is a physical symbol vehicle in a material symbol system. The specific selection of symbols and their syntax (particular sequencing) prescribes needed three-dimensional molecular structures and metabolic cooperative function *prior to* natural selection’s participation. (Source: http://www.genome.jp/dbget-bin/www_bget?refseq+NC_009576).

Figure 3 shows the prescriptive coding of a section of DNA. Each letter represents a choice from an alphabet of four options. The particular sequencing of letter choices prescribes the sequence of triplet codons and ultimately the translated sequencing of amino acid building blocks into protein strings. The sequencing of amino acid monomers (basically the sequencing of their R groups) determines minimum Gibbs-free-energy folding into secondary and tertiary protein structure. It is this three-dimensional structure that provides “lock-and-key” binding fits, catalysis, and other molecular machine formal functions. The sequencing of nucleotides in DNA also prescribes highly specific regulatory micro RNAs and other epigenetic factors. Thus linear digital instructions program cooperative and holistic metabolic proficiency.

Not only are symbol systems used, but a bijection must occur between two independent symbol systems. Bijection (translation; a symbol system to symbol system correspondence) is rule-based, not physical law-based. No cause-and-effect necessity exists in the linking of anticodons, amino acids, tRNAs, and amino acyl tRNA synthetases with codons. The anticodon is located on the opposite end of tRNA from the amino acid. The correspondence between the two languages is arbitrary and abstract. By arbitrary, we do not mean random. Arbitrary means free from physicodynamic determinism. Bijection rules are freely selected. Translation of this linear digital prescription into functionally specific polyamino acid chains cannot be explained by physicodynamics. It is not law-based, and it certainly is not random. If this were an empirical/inductive contention, “cannot” would have to be replaced with “has not yet been.” The problem is that the statement is a valid inference of deductive logic. The conclusion is as unequivocal as that produced by balanced mathematical manipulations of any equation. Neither fixed/forced laws nor chance can logically make non trivial computationally halting programming decisions. It is a logical impossibility for chance and/or necessity to exercise bona fide choice contingency. They are in isolated categories (see Section 8). Neither unaided Markov chains nor physicodynamic determinism can select for *potential* formal function.

The noise-reducing Hamming “block coding” of triplets of nucleotides to prescribe each specific amino acid is all the more abstract and formally conceptual. The triplet codon/amino acid coding table has been shown to be conceptually ideal in a formal sense [286]. Block-coding greatly reduces the ill effects of a noisy channel on transmitted messages. Fewer prescriptive reading errors occur. Translation between the nucleotide and amino acid symbol systems is extraordinarily reliable. In addition, organisms possess amazing repair mechanisms to undo what noise pollution effects do occur to biomessages. Physics and chemistry provide no mechanisms to explain any of these sophisticated formal control and correction capabilities. They clearly traverse The Cybernetic Cut [9]—a great divide in nature between those phenomena that can be explained through the chance and necessity of natural process vs. those phenomena that can only be explained through formal steering and controls.

But the peculiarity of life over inanimate physics extends far beyond the above discussion. DNA requires editing in the course of its transcription to coding mRNA. And we have not even touched on the roles of many other independent players in the formal integration of transcription, translation, regulation, metabolism, and development. Epigenetic factors are a large part of overall holistic true organization [287–292]. Post-translational editing also plays a role [293–296].

As physicist Howard Pattee has demonstrated in many publications [191, 192, 297–302], open-ended evolution (OEE) is impossible without a linear digital genetic symbol system that can mutate independent of the real-time living of the phenotypic organisms that harbor them. Outwardly, the same relatively stable phenotypes exist and mate while tremendous modifications can be occurring in their genomes. Says Pattee, “A non-dynamic descriptive model evades an infinite regress by leaving time out of its rules and symbols. Self-describing models interact with dynamical systems by codes that we tacitly understand as writing, reading and interpreting.” . . . . “Separate description and construction components are necessary for complex systems that can adapt and evolve” [303].

Most mutations are silent. Genetic drift would be impossible without a genetic material symbol system (MSS) that can experience abundant variation within the same basic phenotype [196, 304, 305]. The phase space of potential new instructional sequences would be severely limited if genetic drift from successive point mutations, duplications, inversions, transpositions, crossings over, could not progress at the genetic level prior to phenotypic realization.

*Literal genetic algorithms*, not figurative ones, prescribe and control life. Nucleotides function in an objective, not just a human subjective symbolic capacity. The particular symbol selection at each decision node of nucleotide polymerization is isolated from physicodynamic causation by a *dynamic discontinuity* [196, 304, 305]. Although the instructions are physically instantiated into material symbol systems using physical symbol vehicles, the programming is fundamentally formal. “Semantic/semiotic/bioengineering function requires dynamically inert, resortable, physical symbol vehicles that represent time-independent, non-dynamic “meaning.” (e.g., codons).” [1] No empirical or rational basis exists for granting to physics or chemistry such non-dynamic capabilities of functional sequencing. Neither chance nor necessity (fixed law) can program configurable switches to integrate circuits or organize formal utility.

Linear digital prescription in physical nucleic acid has thus far invariably been associated with life. A fully post modern anthropocentrism cannot argue a logically consistent macroevolutionary paradigm. If naturalistic/materialistic science believes anything, it believes that an objectively real “physical brain secretes mind as the liver secretes bile” [as Pierre Jean Georges Cabanis (1757–1808), Karl Vogt and many others since have phrased it]. Jakob Moleschott (1822–1893) is generally given credit for the renal version: “The brain secretes thought as the kidney secretes urine.” For macroevolution theory to fly, a very real genetic symbol system must evolve through objectively real early eukaryotes, invertebrates, vertebrates, mammals and primates. A purely subjective or solipsistic view of nucleotides and codons—trying to deny that they are real physical symbol vehicles—totally compromises macroevolutionary theory.

Macroevolution theory of necessity presupposes a literal history of progressive adaptation of millions of objectively existent species through changes in objectively existent nucleotide symbol sequencing. The formal, representational codon table not only predates human minds, but humans themselves.

## 8. Symbolic dynamics analysis

It is important not to confuse objectively existent Random, Ordered and Functional Sequence Complexities in nature with symbolic dynamics analysis methodologies [306–309] created and applied by human experimenters. The symbolic dynamics models of human minds use abstract symbols to represent each state in discrete time intervals. Evolution is described by infinite sequences of symbols. A sophisticated shift operator must also be used. All aspects of symbolic dynamics, like the scientific method itself, is a formal enterprise, not a physicodynamic cause and effect chain of the inanimate physical world. As pointed out in the introduction of this paper, applied sciences such as symbolic dynamics analysis provide no help in explaining either gene emergence or spontaneous metabolic self-organization.

Both symbolic dynamics and objective genetic cybernetic programming [12] employ linear digital (discretized) symbol strings. In symbolic dynamics, if the state vector is not inherently discrete, it must be discretized to yield what is called a coarse-grained description of the system. But that is about where the similarities end.

In symbolic dynamics, we assign an arbitrary symbol to represent each discrete physicodynamic state. But inanimate nature cannot *represent* anything using symbols. The latter is a formal function, not a physicodynamic effect that would occur in a primordial environment. Second, using a symbol to represent a physicodynamically determined state is not a control function. It is merely a descriptive function similar to symbolizing initial conditions with formal units of measure. Although both are formal functions, neither is cybernetically determinative. The symbol does not represent a *prescriptive decision node choice* from among real options. Thus a sequence of symbols in symbolic dynamics serves no programming function. Linear digital genetic prescription does. The latter programming strings not only predate animal existence, they produced animals, their brain and minds. Such programming cannot be reduced to human epistemological models of information. Genetic cybernetics at the cellular level is objective, not subjective.

In symbolic dynamics, probability distributions of complexity measures are used to describe and analyze chaotic states. This places symbolic dynamics on similar footing with Shannon transmission engineering. Both systems measure probabilistic combinatorialism. Neither can address meaning, function, or the prescription of formal cybernetic function.

In symbolic dynamics (and most Monte Carlo simulations) time is measured in discrete intervals. Genetic symbol systems, like language, are time-independent in the sense that a seed’s genome can remain in a state of suspended animation for centuries, yet still prescribe the same metabolic integration and life. The genome’s messages are meaningful and functional in multiple time frames, environments, and with varying rates of catalysis.

The “words” in symbol sequences of symbolic dynamics analysis are derived and recognized through an arbitrary formal scheme generated by investigators’ minds. This is artificial selection. It has no parallel in natural selection. Natural selection is nothing more than differential survival and reproduction of already-computed phenotypic organisms. Differential survival plays no role in molecular evolution or initial genetic programming.

## 9. Two kinds of contingency

Contingency means that events could have happened other than what unfolded [310]. Outcomes are not fully determined by prior cause-and-effect chains. Variability and degrees of freedom exist. Outcomes are not “necessary”—they are not mandated by natural laws working on initial conditions. But there are two kinds of contingency, 1) Chance contingency and 2) Choice contingency.

1. Chance contingency is exampled by heat agitation and Brownian movement of molecules in gas and fluid phases. We refer to chance contingency as “randomness.” Chance contingency is statistically describable and predictable. Relative degrees of determinism and chance contingency can co-exist. Weighted means can be calculated for situations with seeming incomplete determinism. Some argue that all physical behavior is ultimately caused, and that chance contingency is only an illusion. Combinations of forces and their effects can be extremely complex. Yet-to-be-discovered forces and relationships may also be at work [199]. But functionally, on the macroscopic level especially, distinct advantages obtain from regarding chance contingency as real and for quantifying possible outcomes statistically.
2. Choice contingency obtains at true decision nodes. Decision nodes are much more than mere bifurcation points. Bifurcation points can be traversed by chance contingency. Any attempt to reduce decision nodes to mere bifurcation points results in rapid deterioration of any potential non trivial formal function. The existence of bifurcation points does not account for computational success. Organization and formal utility are achieved through the controlled opening and closing of logic gates. The latter requires bona fide choices made with steering and programming intent.

## 10. Configurable switches

**Figure 4.**
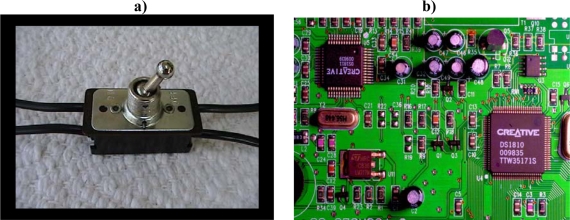
a) A binary configurable switch. Though physical, the switch-setting is nonetheless physicodynamically inert (“dynamically decoupled or incoherent” [196, 197]). No physical force field determines the direction this knob is pushed. The vector of knob push is determined by formal choice contingency alone, not by chance or necessity, and not by order or complexity. b) An integrated circuit board arises only out of unified, coherent, purposefully cooperative, truly organized logic-gate switch-settings. The number of permutations of voluntary (choice-contingent; configurable) switch-setting combinations quickly becomes staggering. Often only one configuration achieves a certain functional computational halting.

Figure 4 shows an old-fashioned binary configurable switch. Such a switch represents the simplest decision node. Everything computational and organizational stems back to binary decision nodes. Binary decision nodes are the basis of all formal function. Even analog and index systems are ultimately based on binary choices. An analog rheostat knob, for example, must be designed to increase power when turned in one direction (e.g., clockwise) and to decrease power when turned in the opposite direction (e.g., counterclockwise).

Can we describe any gradual “degrees of organization” that are possible in the flipping of each binary switch knob? Note that the pictured switch knob cannot be found in a neutral position. The switch is designed with a logical “excluded middle.” It will always be found in either the on or off position. Such configurable switches are designed to record yes/no, on/off, 1/0 purposeful programming choices. There is no gradation of selection at each individual binary decision node. The switch knob will be found in either the right or left position.

Configurable switches are dynamically inert (dynamically incoherent; dynamically decoupled from physicodynamic causation) [196, 197]. This means that on a horizontal switch board, the force of gravity works equally on all potential switch positions. Physicodynamics plays no role in which way the switch knob is pushed. This is the very meaning of “configurable” switches. Their setting is completely decoupled from physicodynamic causation. They can only be set by formal choice contingency, not by chance or law. It is the freedom of formal choice at configurable switches that makes all forms of formal sophistication possible in any physical system. Nonphysical formalism alone determines each switch setting. The switch is a “dynamically-inert configurable switch”.

The switch in Figure 4 happens to be a binary switch. We could have just as easily photographed a quaternary switch. With a quaternary switch, the knob could be pushed away from you, pulled toward you, pushed to the right, or pushed to the left. A quaternary configurable switch represents 2 bits of uncertainty. The option space of equally available four possible nucleotides also represents 2 bits of uncertainty. Each potential add-on locus in a forming single-stranded oligoribonucleotide in an imagined primordial soup adds an additional 2 bits of uncertainty to the strand. The same is true of a single-stranded (positive, instructional) DNA polymer. Each locus corresponds to a four-way (tertiary) configurable switch. The high degree of uncertainty in a potential single-stranded DNA physical matrix is what allows DNA to retain such tremendous amounts of information. Spinelli & Mayer-Foulkes [311] found specific statistical differences between exon and intron DNA sequences, referrring to them as “linguistic DNA features.” Large numbers of other researchers have found linguistic like properties in DNA prescriptive information as summarized by Searls [312].

Although statistical differences and patterns distinguish one linear digital prescriptive string from another, no prescriptive information exists because of probabilistic combinatorialism [77]. Prescriptive information only exists at the moment a particular choice for potential function is made [1]. When a nucleotide is rigidly (covalently) bound to the single-stranded string, the four-way configurable switch knob is actually pushed in one of four possible directions. At that moment all Shannon uncertainty is replaced with formal causation. The vector of the four-way switch knob is determined by choice contingency, not by physicodynamics. It is only when one of the four options is actually selected for potential function that prescriptive information comes into existence. It is only when that choice initiates movement of the physical switch knob in one of the four directions that formalism is instantiated into physicality.

## 11. Two kinds of selection

Two kinds of selection exist: 1) Selection *of existing* function (e.g., natural selection; differential survival) VS. 2) Selection *for potential* function (e.g., artificial selection for formal function).

Selection *of existing* fitness is accomplished by selection pressure. Natural selection consists of differential survival and reproduction of the fittest already-computed phenotypes. It occurs only at the organismic level of already-living small populations of organisms. “Survival of the fittest” is environmental selection of the best existing breeds, varieties, and species.

Selection *for potential* fitness is always artificial rather than natural. Selection for potential fitness is a formal, not a physical enterprise. Selection for potential fitness occurs at decision nodes. Symbols systems and configurable switch settings are used to represent those decisions. Examples of formal selection include language, cybernetic programming, logic, math, computation, algorithmic optimization, design and engineering function, organization of any kind.

Linear digital genetic programming using a Hamming block code of 3 nucleotide selections to represent and prescribe each amino acid selection is a form of selection for potential fitness, not selection of existing fitness. Genetic programming cannot be explained by natural selection. The environment cannot select for potential function. Evolution has no goal or programming ability at the genetic level. As discussed above, the selection of each nucleotide corresponds to the setting of a four-way quaternary configurable switch. Three quaternary switch-settings in a row prescribe each amino acid “letter” of a very long protein “word.” No fitness exists for the environment to favor or select at the level of 3’5’ phosphodiester bond formation between nucleotides. These informational biopolymers must be sequenced prior to the realization of any prescriptive, enzymatic, or regulatory function. Selection at the level of nucleotide sequencing clearly falls within the category of “Selection for potential function” rather than the category of “Selection of existing function.” This is called the GS (Genetic Selection) Principle [10]. The GS Principle states that selection must occur at the decision-node level of rigid covalent bond linkage of specific monomers to form functional syntax. After-the-fact selection of already computed phenotypic fitness is not sufficient to explain genetic programming or the metabolism it organizes.

We must also remember that natural selection does not favor function. Selection pressure favors only the survival of the fittest holistic, already-living organisms. No organism would be alive without thousands of cooperating molecular machines, integrated biochemical pathways and cycles, and the formal goal of maintaining a homeostatic metabolism. All of these algorithmic processes must be optimized and in place before any organism can come to life, let alone constitute the fittest selectable life. Chang *et al.* [313] state:

‘Chemical evolution’ should not be confused with Darwinian evolution with its requirements for reproduction, mutation and natural selection. These did not occur before the development of the first living organism, and so chemical evolution and Darwinian evolution are quite different processes.

## 12. What optimizes genetic algorithms?

Computational methods often employ genetic algorithms (GA’s). The appeal of GAs is that they are modeled after biological evolution. The latter is the main motivation for tolerating such an inefficient awkward process. The GA search technique begins with a large random pool of representations of “potential solutions.” Genetic algorithms are seen as a subset of evolutionary algorithms and as “evolutionary computation.” The methodology is inspired by modeling a random beginning phase space, various kinds of mutations, inheritance and selection. The experimenter chooses the fittest solutions from each generation out of the “evolving” phase space of potential solutions. The goal of the process is optimization of a certain function.

All too many evolutionary computationists fail to realize the purely formal nature of GA procedures. GA’s are not dealing with physicodynamic cause-and-effect chains. First, what is being optimized is *a formal representation* of meaning and function. A representation of any kind cannot be reduced to inanimate physicality. Second, “potential solutions” are formal, not merely physical entities. Third, at each iteration (generation) a certain portion of the population of potential solutions is deliberately selected by the agent experimenter (*artificial selection*) to “breed” a new generation. The optimized solution was purposefully pursued at each iteration. The overall process was entirely goal-directed (formal). Real evolution has no goal [172–175]. Fourth, a formal fitness function is used to *define* and *measure* the fittest solutions thus far to a certain formal problem. The act of defining and measuring, along with just about everything else in the GA procedure, is altogether formal, not physical [140, 194, 298, 314, 315].

Despite the appealing similarities of terms like “chromosomes,” GA’s have no relevance whatsoever to molecular evolution or gene emergence. Inanimate nature cannot define a fitness function over measures of the quality of representations of solutions. GAs are no model at all of natural process. GA’s are nothing more than multiple layers of abstract conceptual engineering. Like language, we may start with a random phase space of alphabetical symbols. But no meaning or function results without deliberate and purposeful selection of letters out of that random phase space. No abiotic primordial physicodynamic environment could have exercised such programming prowess. Neither physics nor chemistry can dictate formal optimization, any more than physicality itself generates the formal study of physicality. Human epistemological pursuits are formal enterprises of agent minds. Natural process GAs have not been observed to exist. The GAs of living organisms are just metaphysically presupposed to have originated through natural process. We can liberally employ GAs and so-called evolutionary algorithms for all sorts of productive tasks. But GAs cannot be used to model spontaneous life origin through natural process because GAs are formal.

## 13. Order vs. Organization

Organization ≠ order. Disorganization ≠ disorder. Self-ordering of many kinds occurs spontaneously every day in nature in the absence of any organization. Spontaneous bona fide self-organization, on the other hand, has never been observed.

“Self-organization” is logically a nonsense term. Inanimate objects cannot organize themselves into integrated, cooperative, holistic schemes. Schemes are formal, not physical. To organize requires choice contingency, not just chance contingency and law-like necessity. Sloppy definitions lead to fallacious inferences, especially to category errors. Organization requires 1) decision nodes, 2) steering toward a goal of formal function, 3) algorithmic optimization, 4) selective switch-setting to achieve integration of a circuit, 5) choice with intent.

The only entity that logically could possibly be considered to organize itself is an agent. But not even an agent self-organizes. Agents organize things and events in their lives. They do not organize their own molecular biology, cellular structure, organs and organ systems. Agents do not organize their own being. Agents do not create themselves. They merely make purposeful choices with the brains and minds with which they find themselves. Artificial intelligence does not organize itself either. It is invariably programmed by agents to respond in certain ways to various environmental challenges in the artificial life data base.

Thus the reality of self-organization is highly suspect on logical and analytic grounds even before facing the absence of empirical evidence of any spontaneous formal self-organization. Certainly no prediction of bona fide *self*-organization from unaided physicodynamics has ever been fulfilled. Of course if we fail through sloppy definitions to discern between self-ordering phenomena and organization, we will think that evidence of self-organization is abundant. We will point to hundreds of peer-reviewed papers with “self-organization” in their titles. But when all of these papers are carefully critiqued with a proper scientific skepticism, our embarrassment only grows with each exposure of the blatant artificial selection that was incorporated into each paper’s experimental design. Such investigator involvement is usually readily apparent right within Materials and Methods of the paper.

## 14. What exactly is chaos?

Chaos is a bounded state of *disorganization* that is extremely sensitive to the effects of initial conditions. Note that chaos is a disorganized state of matter, *not a disordered* state of matter. A considerable amount of order can arise spontaneously out of chaos. This is what chaos theory is about. Prigogine’s dissipative structures are rapid successions of momentarily self-ordered states. Chaos theory deals with such spontaneously forming forms and order. All we have to do to observe spontaneous self-ordering is to pull the stopper out of our bathtub drain. Water molecules quickly self-order into a swirl—a vortex—from purely physicodynamic complex causation. We mistakenly call this self-organization. The vortex is not organized. It is only self-ordered [4]. What is the difference? No decision nodes are required for a bathtub swirl to self-order out of seemingly random Brownian movement. Proficient programming choices are not required for heat agitation of water molecules to self-order into a vortex. No configurable switches have to be purposefully set, each in a certain way, to achieve self-ordering. No pursuit of a goal is involved. No algorithmic optimization is required. In addition, Prigogine’s dissipative structures do not DO anything formally productive. They possess no ability to achieve computational halting.

Chaos is capable of producing incredibly complex physicodynamic behavior. But we must never confuse this complexity with formal function. The shape of a candle flame is a spontaneously self-ordered shape or form. It is a rapid succession of dissipative structures that creates the illusion of a sustained structure. Order spontaneously appears out of disorder in the complete absence of any formal creative input or cybernetic management. But no algorithmic organization is produced by a candle flame. The sustained shape of a candle flame is self-ordered. It is not self-organized [4].

The dissipative structures of Prigogine arise out of high-order cause-and-effect “necessity.” What seems to be a totally random environment is in fact a caldron of complex interaction of multiple force fields. The complexity of interactive causation can create the illusion of randomness, or of very real self-ordering. There may also be as-of-yet undiscovered physical causes. But dissipative structures self-order; they do NOT self-organize. The dissipative structures of chaos theory are unimaginative. Highly ordered structures contain very little information. Information retention in any physical medium requires freedom of selection of configurable switch settings. Switches must be “dynamically inert” with respect to their function as decision nodes. Dissipative structures are

1. highly ordered,
2. monotonous,
3. predictable,
4. regular (vortices, sand piles)
5. low informational
6. strings of momentary states

Dissipative structures are usually destructive, not cybernetically constructive (e.g., tornadoes, hurricanes). Trying to use “chaos” and “complexity” to provide mechanism for “self-organization” is like trying to use the Shannon transmission engineering to explain intuitive information, meaning and function. Shannon’s equations define “surprisal” and “uncertainty,” not semantic information. Just as we cannot explain and measure “intuitive information” using Shannon combinatorial uncertainty, we cannot explain a truly organized system appealing to nothing but a mystical edge of chaos. Reduced uncertainty (“mutual entropy”) in Shannon theory comes closer to semantic information, but only because we mix in the formal elements of human knowledge. We measure the reduced uncertainty of *our knowledge*. At that point, we are no longer talking about objective information in nature. We are only talking about human epistemology. Human consciousness is highly subjective. The second we insist on defining information solely in terms of a human observer, we have destroyed all hope of elucidating the derivation of objective information in evolutionary history.

The disorganization of chaos is characterized by conceptual uncertainty and confusion. Disorganization lacks sophisticated steering and control. Disorganization pursues no purpose. Even if chaos had a purpose, it would lack all means of accomplishing that purpose. If chaos by definition is a bounded state of disorganization, how could we possibly attribute self-organization to chaos? No scientific basis exists for granting formal capabilities to chaos, complexity or catastrophe. None of these three has ever been observed to produce formal integration and algorithmic organization of any kind.

Scientists accomplish impressive feats using nonlinear dynamics. But our use of the phrase “nonlinear dynamics” all-too-easily starts referring to chaos as though chaos itself were capable of achieving formal function. We overlook the considerable degree of “investigator involvement” and artificial steering that went into nonlinear dynamic experiments. Formal mathematics was invariably employed by agents. No observers or knowers would exist were it not for a phenomenal amount of *objective* information instructing each cell. A great deal more objective prescriptive information is required to integrate cell systems, organs, organ systems, and holistic organisms. No observers or knowers were around when bacteria were being prescribed and their initial instruction sets being replicated and reproduced. Human observers are Johnny-come-lately *discoverers* of information. Human epistemology is not an essential component of what objective genetic prescriptive information *is* in nature.

Many scientists across a wide array of disciplines exercise a surprisingly blind faith in the amazing formal capabilities of spontaneous molecular chaos and combinatorial complexity. Empirical and rational support for this belief system is sorely lacking. Achieving sophisticated formal function consistently requires regulation and control. Control always emanates from choice contingency and intentionality, not from spontaneous molecular chaos.

## 15. The Edge of Chaos

If chaos is inadequate to explain self-organization, what about “the Edge of Chaos?” [7, 8, 21, 22, 50–57, 198, 316–328] The edge of chaos is somehow much more appealing to us than just plain chaos. The edge of chaos is more poetic. It is terribly-inviting. It offers much more mystical allure. The question is, does the edge of chaos actually exist? If the edge of chaos is objectively real, what exactly is it? Where in time/space can we find it, and what can it independently do? Is the edge of chaos even scientifically addressable?

Let us first examine the potential interface of chaos with natural order—with the regularities of nature described by the physical laws. Can “order” program configurable switches? If “order” programmed configurable switches, they would all be programmed the same way. They would all be set to “On’s,” OR. . . they would all be set to “Off’s.” Either way, the configurable switches would not be formally programmed into any algorithmic function. No more creativity would exist at the interface of bounded disorganization with forced order than in either single entity. No reason exists to expect any increased cybernetic potential at the edge of chaos than squarely in the middle of chaos (bounded disorganization). The fact that chaos is extremely sensitive to the effects of initial conditions adds no formal attributes. The latter certainly increases its changeability and the number of bits of uncertainty in the bounded state. But mere changeability and combinatorial uncertainty provide no optimization of formal function.

What about the interface of the bounded state of disorganization with heat agitation and Brownian movement? Maximum complexity would set all configurable switches randomly. What synergistic capabilities could emerge from the interface of disorganization with randomness? The two are not synonymous. But neither contributes anything to programming proficiency.

What scientific substance does the edge of chaos provide? What empirical support do we have of formal function arising spontaneously from the interface of chaos with chance OR necessity? What is the logic behind such anticipation? What empirical support do we have for the computational proficiency of the edge of chaos? Have we had any prediction fulfillments since it was first described in 1992 by Waldrop [50]? Is the notion of vast formal capabilities arising from the edge of chaos falsifiable? One has to wonder if the notion is worthy of serious discussion in a peer-reviewed science journal paper. It would not be were it not for the fact that so many peer-reviewed papers already cite this nebulous dream as an objective source of self-organization.

## 16. Systems theory

Systems theory in the literature regularly presupposes the metaphysical belief of physicodynamic self-organization into formal function. One would think that systems theorists could readily offer a crystal-clear definition of “system.” Sadly, this is not the case. It is not surprising, therefore, that chaos and such phenomena as weather fronts are referred to as systems with no eyebrows raised. Bona fide systems require organizational controls. True systems are cybernetic. Weather fronts are at best self-ordered by complex degrees of interactive physicodynamic causation. They are not formally controlled or organized to achieve sophisticated utility of any kind. A weather front is a physicodynamic interface complete with criticality and phase changes. It may become a highly self-ordered tornado or a hurricane. But it’s not a true system because it is not formally organized or cybernetically programmed. No representational symbol system is used. No abstract conceptualizations are employed by weather fronts. They are simply physicodynamic interfaces totally lacking in algorithmic organization. We simply “murder the King’s English” by referring to a weather front as a system. Such sloppy word usage leads to a great deal of confusion in understanding fundamental physics, the temporary and local circumvention of the 2nd Law, and the algorithmic processes that alone make the latter possible.

Chaos is neither organized nor a true system, let alone “self-organized.” As pointed out above, organization is not the same as order. A bona fide *system* requires *organization*. Chaos by definition lacks organization. That’s why we call it “chaos” even though it manifests extensive self-ordering tendencies. What could possibly be more self-ordered than a massive hurricane? But what formal functions does it perform? A hurricane doesn’t DO anything constructive or formally functional because it contains no formal organizational components. It has no programming talents or creative instincts. A hurricane is not a participant in Decision Theory. A hurricane does not set logic gates according to arbitrary rules of inference. A hurricane has no specifically designed dynamically-decoupled configurable switches. No means exists to instantiate formal choices or function into physicality. A highly self-ordered hurricane does nothing but destroy organization. To call a hurricane “self-organized” constitutes one of the most egregious errors in science stemming from sloppy definitions, category errors, and non sequiturs.

In itself, chaos is NOT a

1. Calculus.
2. Algorithm.
3. Program that achieves computational halting.
4. Organizer of formal function.
5. A bona fide system.

Complexity is not a system, either, as we saw in the highly complex pile of pick-up sticks (Figure 2). No programming is involved. No algorithms are optimized. No steering toward formal function occurs. A true system requires organization.

In physics, no empirical evidence exists, not even an anecdotal account, of Chaos, Catastrophe, maximum Complexity, order or pattern ever having produced sophisticated algorithmic function or cybernetic organization of any kind. A pulsar signal has abundant order and pattern. But it doesn’t DO anything useful. It contains no meaningful or functional message. It knows nothing of decision nodes or choice contingency.

In biology, no rational or empirical justification exists for attributing linear, digital, encrypted, genetic recipes to stochastic ensembles OR to physical laws in *any* amount of time. Yet thousands of peer-reviewed papers exist in the literature on “self-organization.” How can denial of self-organization possibly be correct? The answer is that all of these papers are universally misdefining what is being observed. Self-ordering phenomena are being observed, not self-organization. But self-ordering phenomena do not measure up to the task of genetic programming.

## 17. Formalism vs. Physicality

When it comes to life-origin studies, we have to address how symbol selection in the genetic material symbol system came about objectively in nature [2]. Life origin science must address the derivation of objective organization and control in the first protocells. How did prescriptive information and control arise spontaneously out of the chaos of a Big Bang explosion, primordial slime, vent interfaces in the ocean floor, or mere tide pools?

Self-ordering phenomena arise spontaneously out of phase space, but we have no evidence whatsoever of formal organization arising spontaneously out of physical chaos or self-ordering phenomena. Chance and necessity has not been shown to generate the choice contingency required to program computational halting, algorithmic optimization, or sophisticated function.

If chance and necessity, order and complexity cannot produce formal function, what does? *Selection for potential* utility is what optimizes algorithms, not randomness (maximum complexity), and not fixed law (highly patterned, unimaginative, redundant order). Utility lies in a third dimension imperceptible to chance and necessity. What provides this third dimension is when each token in a linear digital programming string is arbitrarily (non physicodynamically, formally) selected for potential function. The string becomes a cybernetic program capable of computation only when signs/symbols/tokens are arbitrarily *chosen* from an alphabet to *represent* utilitarian configurable switch settings. The choice represented by that symbol can then be instantiated into physicality using a dynamically inert (physicodynamically decoupled or incoherent) [196, 197, 329] configurable switch setting. At the moment the switch knob seen in Figure 4 is pushed, nonphysical formalism is instantiated into physicality. Then and only then does algorithmic programming become a physical reality. Once instantiated, we easily forget the requirement of instantiation of *formal instructions and controls* into the physical system to achieve engineering function. It was the formal voluntary pushing of the configurable switch knob in a certain direction that alone *organized* physicality [1, 3, 4, 7–9, 12].

Degrees of integration are achieved through *a combination* of binary configurable switch-settings. The selection of any combination of multiple switch settings to achieve degrees of organization is called programming. But purposefully flipping the very first binary configurable switch is the foundation and first step of any form of programming. Programming requires choice contingency. The measure of algorithmic compression requires an added dimension. Only this extra dimension allows us to place a sequence on the unidimensional vector graph showing varying degrees of order and complexity (Figure 1). No known natural process spontaneously compresses an informational message string. As Howard Pattee has repeatedly pointed out, any type of measurement is a formal function that cannot be reduced to physicodynamics [161, 314, 330, 331]. We do not plug initial conditions into the formal equations known as “the laws of physics.” We plug *symbolic representations* of those initial conditions into the laws of physics. Then we do formal mathematical manipulations of these equations to reliably predict physicodynamic interactions and outcomes. In this sense formalism governs physicality. The role that mathematics plays in physics is alone sufficient to argue for formalism’s transcendence over physicality.

Just as it takes an additional dimension to measure the algorithmic compressibility of a sequence, it takes still another dimension to measure the formal utility of any sequence. Formalisms are abstract, conceptual, representational, algorithmic, choice-contingent, non physical activities of mind. Formalisms typically involve steering toward utility.

Formalisms employ controls rather than mere physicodynamic constraints. Formalisms require obedience to arbitrarily prescribed rules rather than forced laws. Physicodynamics cannot visualize, let alone quantify formal utility. Formalisms cannot be produced by chance or necessity. Language, for example, uses arbitrary symbol selections from an alphabet of options. Logic theory uses rules, not laws, to judge inferences. Programming requires choice contingency at each decision node. Each logic gate and configurable switch must be deliberately set a certain way to achieve potential (not-yet-existent) computational halting. These are all formal functions, not spontaneous physicodynamic events. They are just as formal as mathematics. Decision nodes, logic gates, and configurable switches cannot be set by chance and/or necessity if sophisticated formal utility is expected to arise. They must be set with the intent to control and to program computational halting. Acknowledgement of the reality of formal controls was growing within the molecular biological community even prior to the now weekly new discoveries of extraordinarily sophisticated cybernetic mechanisms in cellular physiology [332].

## 18. The Cybernetic Cut

Formal function can invariably be traced back to the exercise of some form of decision theory. Achieving formal utility requires crossing The Cybernetic Cut [9]. The Cybernetic Cut is perhaps the most fundamental divide of scientifically addressable reality. A monstrous ravine runs through presumed objective reality. It is the great divide between physicality and formalism. On the one side of this Grand Canyon lies everything that can be explained by the chance and necessity of physicodynamics. On the other side lies those phenomena than can only be explained by formal choice contingency and decision theory—the ability to choose with intent what aspects of ontological being will be preferred, pursued, selected, rearranged, integrated, organized, preserved, and used. Physical dynamics includes spontaneous non linear phenomena, but not our formal applied-science called “non linear dynamics”.

A configurable-switch (CS) Bridge traverses this great chasm. But this CS Bridge conveys one-way traffic only. Prescriptive information flows only from the formal side to the physical side of the ravine. Programming decisions can be instantiated into physical configurable switch settings. But physicality contributes no formal influence on those choices in reverse direction. The choices that set the physical configurable switches are themselves non physical. Physicodynamic forces have no influence upon non physical formalisms. Physicodynamics (the chance and necessity of physicality) cannot steer events toward computational halting.

Falsification of The Cybernetic Cut requires nothing more than demonstrating a single incident of two-way traffic across the CS Bridge. Thus far, no such incident of two-way traffic has even been observed. Logically, the chance and necessity of physicality cannot make purposeful choices (e.g., programming decisions). Physicality cannot plot and scheme. Physicality cannot prefer utility over non utility. It cannot even categorize formal function from non function. Stand-alone physicodynamics is blind to utility, and could care less whether anything “works” in a formal sense.

This is not to say that formalisms cannot employ elements of chance and/or physicodynamic determinism. Every day architects and engineers work around, depend upon, and deliberately employ the orderliness of physicodynamics. When we play the card game of poker, we incorporate stochastic reality and physical constraints into our formal scheming. These facts in no way threaten the reality of The Cybernetic Cut.

The Cybernetic Cut is logically, not empirically, absolute. Science does not expect induction to be absolute. But within any axiomatic deductive system, when the rules of inference are carefully adhered to, we have every right to draw as firm a conclusion as we do when predicting physical interactions with any mathematical law of physics. If the predictions fail, we have reason to question our initial axiomatic presuppositions. Thus far, we have no reason or empirical evidence that would cause us to doubt the axiom of The Cybernetic Cut and the one-way traffic across its CS Bridge.

In those fields relating to non linear dynamics, we tend to point to chaos theory, complexity theory, fractals, rugged fitness landscapes, Markov chains, evolutionary algorithms, and directed evolution as evidence for the self-organization of physicality. In reality, all of these fields and models serve only to reinforce the reality of The Cybernetic Cut. Investigator involvement (various forms of artificial selection, not natural selection) is readily identifiable in hundreds of these published experimental designs. A classic example is the body of published ribozyme engineering experiments [333–335]. Take away the experimenter’s purposeful choosing of which iteration to pursue, and the desired ribozyme devolves every time toward either a non functional stochastic ensemble, or a self-ordered polymer such as a polyadenosine that also does nothing useful. Neither chance nor necessity can program the needed ribozyme.

## 19. Conclusions

The capabilities of stand-alone chaos, complexity, self-ordered states, natural attractors, fractals, drunken walks, complex adaptive systems, and other subjects of non linear dynamic models are often inflated. Scientific mechanism must be provided for how purely physicodynamic phenomena can program decision nodes, optimize algorithms, set configurable switches so as to achieve integrated circuits, achieve computational halting, and organize otherwise unrelated chemical reactions into a protometabolism. To focus the scientific community’s attention on its own tendencies toward overzealous metaphysical imagination bordering on “wish-fulfillment,” we propose the following readily falsifiable null hypothesis, and invite rigorous experimental attempts to falsify it:

“Physicodynamics cannot spontaneously traverse The Cybernetic Cut [9]: physicodynamics alone cannot organize itself into formally functional systems requiring algorithmic optimization, computational halting, and circuit integration.”

A single exception of non trivial, unaided spontaneous optimization of formal function by truly natural process would falsify this null hypothesis.

## Figures and Tables

**Table 1.** FSC of Selected proteins. Supporting data from the lab of Kirk Durston and David Chiu at the University of Guelph [77] showing the analysis of 35 protein families.

	Length (aa)	Number of Sequences	Null State (Bits)	FSC (Fits)	Average Fits/Site
Ankyrin	33	1,171	143	46	1.4
HTH 8	41	1,610	177	76	1.9
HTH 7	45	503	194	83	1.8
HTH 5	47	1,317	203	80	1.7
HTH 11	53	663	229	80	1.5
HTH 3	55	3,319	238	80	1.5
Insulin	65	419	281	156	2.4
Ubiquitin	65	2,442	281	174	2.7
Kringle domain	75	601	324	173	2.3
Phage Integr N-dom	80	785	346	123	1.5
VPR	82	2,372	359	308	3.7
RVP	95	51	411	172	1.8
Acyl-Coa dh N-dom	103	1,684	445	174	1.7
MMR HSR1	119	792	514	179	1.5
Ribosomal S12	121	603	523	359	3.0
FtsH	133	456	575	216	1.6
Ribosomal S7	149	535	644	359	2.4
P53 DNA domain	157	156	679	525	3.3
Vif	190	1,982	821	675	3.6
SRP54	196	835	847	445	2.3
Ribosomal S2	197	605	851	462	2.4
Viral helicase1	229	904	990	335	1.5
Beta-lactamase	239	1,785	1,033	336	1.4
RecA	240	1,553	1,037	832	3.5
tRNA-synt 1b	280	865	1,210	438	1.6
SecY	342	469	1,478	688	2.0
EPSP Synthase	372	1,001	1,608	688	1.9
FTHFS	390	658	1,686	1,144	2.9
DctM	407	682	1,759	724	1.8
Corona S2	445	836	1,923	1,285	2.9
Flu PB2	608	1,692	2,628	2,416	4.0
Usher	724	316	3,129	1,296	1.8
Paramyx RNA Pol	887	389	3,834	1,886	2.1
ACR Tran	949	1,141	4,102	1,650	1.7
Random sequences	1000	500	4,321	0	0
50-mer polyadenosine	50	1	0	0	0

Shown are sequence lengths (column 1), the number of sequences analyzed for each family (column 2), the Shannon uncertainty of the Null State *H_ø_* (the absence of any physicodynamic constraints on sequencing: dynamically inert stochastic ensembles) for each protein (column 3), the FSC value ζ in Fits for each protein (column 4), and the average Fit value/site (FSC/length, column 5). For comparison, the results for a set of uniformly random amino acid sequences (RSC) are shown in the second from last row, and a highly ordered, 50-mer polyadenosine sequence (OSC) in the last row. All values, except for the OSC example, which was calculated from the constrained ground state required to produce OSC, were computed from the null state. The Fit values obtained can be discussed as the measure of the change in functional uncertainty required to specify any functional sequence that falls into the given family being analyzed. (Used with permission from Durston, K.K.; Chiu, D.K.; Abel, D.L.; Trevors, J.T. Measuring the functional sequence complexity of proteins. *Theor Biol Med Model* 2007, *4*, Free on-line access at http://www.tbiomed.com/content/4/1/47).
